# Honey bee maternal effects improve worker performance and reproductive ability in offspring

**DOI:** 10.3389/fcell.2023.1156923

**Published:** 2023-04-25

**Authors:** Longtao Yu, Xujiang He, Xinxin Shi, Weiyu Yan, Xiaobo Wu

**Affiliations:** ^1^ Honeybee Research Institute, Jiangxi Agricultural University, Nanchang, China; ^2^ Jiangxi Province Key Laboratory of Honeybee Biology and Beekeeping, Nanchang, China; ^3^ Institute of Insect Sciences, College of Agriculture and Biotechnology, Zhejiang University, Hangzhou, China

**Keywords:** honey bee, maternal effect, queen quality, foraging performance, reproductive ability

## Abstract

Maternal effects are an evolutionary strategy used to improve offspring quality. In an example of maternal effects in honey bees (*Apis mellifera*), mother queens produce larger eggs in queen cells than in worker cells in order to breed better daughter queens. In our current study, morphological indexes, reproductive tissues, and the egg-laying ability of newly reared queens reared with eggs laid in queen cells (QE), eggs laid in worker cells (WE), and 2-day-old larvae in worker cells (2L) were evaluated. In addition, morphological indexes of offspring queens and working performance of offspring workers were examined. The thorax weight, number of ovarioles, egg length, and number of laid eggs and capped broods of QE were significantly higher than those of WE and 2L, indicating that the reproductive capacity of QE group was better than that of other groups. Furthermore, offspring queens from QE had larger thorax weights and sizes than those from the other two groups. Offspring worker bees from QE also had larger body sizes and greater pollen-collecting and royal jelly-producing abilities than those of other two groups. These results demonstrate that honey bees display profound maternal effects on queen quality that can be transmitted across generations. These findings provide a basis for improving queen quality, with implications in apicultural and agricultural production.

## 1 Introduction

Maternal effects, which occur in animals and plants, refer to the effects of the environment (uterine environment, nutrition, light, temperature, etc.) and behavior (laying behavior, breastfeeding, etc.) of parents on offspring phenotypes and are an important mechanism underlying adaptive phenotypic plasticity (Roach and Wulff, 1987; Bernardo, 1996; Moussear and Fox, 1998; Cunningham and Russell, 2000; Wolf and Wade, 2009; Van Dooren et al., 2016; Schwabl and Groothuis, 2019). In rats and hens, the diet of mothers is related to offspring quality (Hseuh et al., 1973; Rehfeldt et al., 2004). In addition, eusocial insects can adjust their investment into eggs via maternal effects in order to adapt to the external environment. For example, queens of ant (*Pheidole pallidula*) and honey bee can selectively lay larger eggs for high-quality offspring (Passera, 1980; Wei et al., 2019). It has also been reported that *Pogonomyrmex* queens are able to determine the developmental fate of their eggs, and levels of ecdysteroids are significantly lower in eggs developing into queens than in those developing into workers (Passera, 1980; Schwander et al., 2008). This is determined only by queens, indicating that queens may strongly influence caste determination in female offspring. We previously detected maternal effects on honey bee quality, in which eggs laid by mother queen were larger in queen cells than in worker cells, resulting in high-quality daughter queens (Wei et al., 2019; He et al., 2021). We also found that queens reared with eggs from queen cells had heavier bodies, more ovarioles, and different gene expression and DNA methylation levels when compared to queens reared with eggs or small larvae from worker cells (Wei et al., 2019; He et al., 2021). Therefore, honey bee maternal effects are vital for queen development and whole colony fitness.

Honey bees are generally important insects, providing abundant products for human use and pollinating plants as an ecosystem service. Most crops are dependent on pollination by honey bee, and honey bee pollination services have been estimated at USD 11.68 billion in the United States (Khalifa et al., 2021). However, bee populations have begun to decline dramatically in many counties (Stankus, 2008; Ratnieks and Carreck, 2010). At present, the factors contributing to the decline of honey bee colonies is still not clear. However, queens with poor quality have been considered as a major reason for the mass death of honey bees (van Engelsdorp et al., 2011; Lee et al., 2019).

Current domestic rearing techniques involve the use of artificial queen cells containing female eggs or larvae that have been placed in a recently queen-less colony and reared as new queens. Due to the difficulties associated with grafting eggs, the grafting of young larvae has become a common approach in commercial queen rearing. Traditional queen rearing technology is widely used in the artificial rearing of queens and involves transplanting young worker larvae. However, queens reared with worker larvae differ from the queens in natural honey bee colonies, and the quality of queens obtained via transfer of older larvae technology is poor. We previously confirmed that this technology continuously reduces queen quality, alters gene expression levels, and increases global DNA methylation levels (Yi et al., 2020).

High-quality queens are important for the development of a colony and the production of honeybee products (Hatjina et al., 2014). Studies have indicated that the survival and reproduction of a bee colony requires more healthy workers which is related to queen reproductive capacity to collect food. Thus, a high-quality queen may produce more workers, which will result in the colony being more productive. We hypothesize that honey bee maternal effects may improve queen quality as well as the fitness of the whole colony. In traditional queen rearing, the development of queens could be profoundly influenced by many factors, such as the grafted larval age and maternal effects (Woyke, 1971; Rangel et al., 2013). Related studies have revealed that the quality of reared queens with young larvae is lower than that of queens reared with eggs, and the quality of queens decreases with an increase in the age of larvae (Woyle, 1971; Rangel et al., 2013). It has been reported that the growth of colonies with queens reared from relatively old larvae is significantly slower than that of colonies with queens reared with younger larvae (Rangel et al., 2013). Furthermore, our previous study found that the offspring queens of high-quality queens had a higher performance of reproductive potential (Yu et al., 2022). However, it is still unclear how maternal effects influence the reproductive ability of offspring queens and foraging capacity of offspring workers. Therefore, in this study, queens were reared with three kinds of eggs or larvae (eggs in queen cells, eggs in worker cells, and 2-day worker larvae) for a comparison of the quality and reproductive potential of daughter queens. The capacity for pollen collection and royal jelly production of offspring workers was also evaluated.

## 2 Materials and methods

### 2.1 Honeybee colony and queen rearing

The colonies of honey bee (*Apis mellifera*) were kept at Jiangxi Agricultural University, Nanchang, China.

In this experiment, we used three different naturally mated queens to generate each a replicate set of QE, WE, and 2L daughter queens, reared from queen eggs (QE), worker eggs (WE) and from second instar larvae (2L). Hence, we had three independent replicates, each consisting of one QE, WE, and 2L queen to build the respective colonies. In order to obtain QE queens, each of the naturally mated source queen was confined on plastic queen cells for egg laying for 12 h. The methods for controlling queen laying eggs on queen cells have been previously reported (Wei et al., 2019). Approximately 64 eggs were laid in queen cells and were transferred to a colony for new queen rearing. The same mother queen was also maintained on a frame of worker cells in order to lay eggs for 12 h. Approximately 128 eggs were laid in worker cells, and 64 of those eggs were transferred to queen cells in order to rear WE queens. The remaining eggs in worker cells were kept in their native colony and hatched to 2-day-old larvae, which were then transferred to queen cells in order to rear 2L queens. Workers delivered enough royal jelly to larvae in those queen cells, which were kept in the same non-experimental colony until all of the queen cells were capped. From the 15th day after laying, these queen cells were transferred to an incubator (35°C, 75% relative humidity) until emergence. The queen cells were checked every 2 h in order to see if any queens had emerged and were then checked hourly after the first queen had emerged. In this experiment, queens of QE, WE, and 2L in different replicant were from different naturally mated queen, and a replicant contained one QE, WE, and 2L queen. A total of 3 different naturally mated maternal queens were used for producing queen of QE, WE, and 2L, and queens of QE, WE, and 2L in the same replicant were from a single maternal queen.

### 2.2 Morphological quality of queens

Approximately 20 queens emerged from each group of queen cells and the thorax weight of those queens was measured. Some of emerged queens from each group were then transferred to queen cages and kept in a non-experimental queen-less colony for 5 days, and they could be fed and maintained by workers through the cage. 5 days later, we dissected the abdomens of virgin queens and harvested the right ovary of each sampled queen to produce paraffin sections and subsequently score the number of ovarioles. The methods for producing paraffin sections and scoring ovariole numbers have been previously described (Yi et al., 2020).

### 2.3 Reproductive quality of queens

QE, WE, and 2L queens were obtained from the same mother queen, as mentioned above, and were kept in mating colonies to mate naturally. Workers (1,000 g) were used to rebuild a new colony. New colonies were then rebuilt and divided into three groups, and successfully mated queens were added to the new colonies separately. After the queens began to lay eggs stably, queens from QE, WE, and 2L were allowed to lay eggs in empty combs from 8:00 to 14:00 every day for 6 days, and the eggs in combs were subsequently counted. In addition, two empty frames were added to beehives, and queens were allowed to lay eggs on these frames. Thereafter, the number of capped cells in each group was recorded every 12 days after the newly mated queens started laying eggs, and records were repeated four times in a row.

### 2.4 Quality of offspring worker bees

Queens reared from QE, WE, 2L were mated and placed on empty combs as explained in above as part 2.3 for 2 h separately. Next, the eggs laid by those newly reared mated queens from each group were collected and their weight and size were measured. When eggs hatched into 1-day-old larvae, they were then transferred to artificial queen cells for royal jelly harvesting in their native colonies. Two days later, 3-day-old larvae were picked out, and the royal jelly in queen cells from each group was weighed.

A capped brood frame from each mating colony, where reared queens had mated and laid eggs, was removed and transferred to an incubator for emergence. Newly emerged worker bees were weighed, and then approximately 500 worker bees in each group were marked with different colors and added to the same colony. On day 26 (after worker bee emergence), forager bees from each group with different color markers were captured at the beehive entrance, the pollen cluster was stripped and weighed. Additionally, the width and length of the left wings of forager bees from each group were measured. However, when we conducted a part of the experiment, one queen bee died, so there was a missing repetition later.

### 2.5 Quality of offspring queens

**FIGURE 1:**
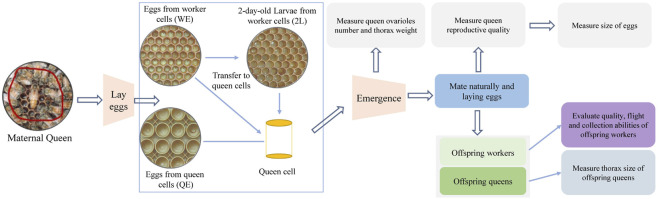
The flow chart of the experiments.

The queens reared from QE, WE, 2L were placed on empty combs for 2 h separately, and the eggs laid by queens in each group were kept in the same non-experimental colony. Four days later, these eggs were hatched to 2-day-old larvae, which were then transferred to new queen cells and reared in the same colony. When offspring queens emerged, the weight and thorax size of newly emerged offspring queens were determined. The flow chart of the experiments was shown in Figure 1.

### 2.6 Statistical analysis

Statistical analyses were performed using SPSS Statistics version 26. All differences among the three groups were determined by one-way ANOVA, and Fisher’s LSD tests were used to determine pairwise differences between groups.

## 3 Results

### 3.1 Number of ovarioles and thorax weight of reared queens

**FIGURE 2:**
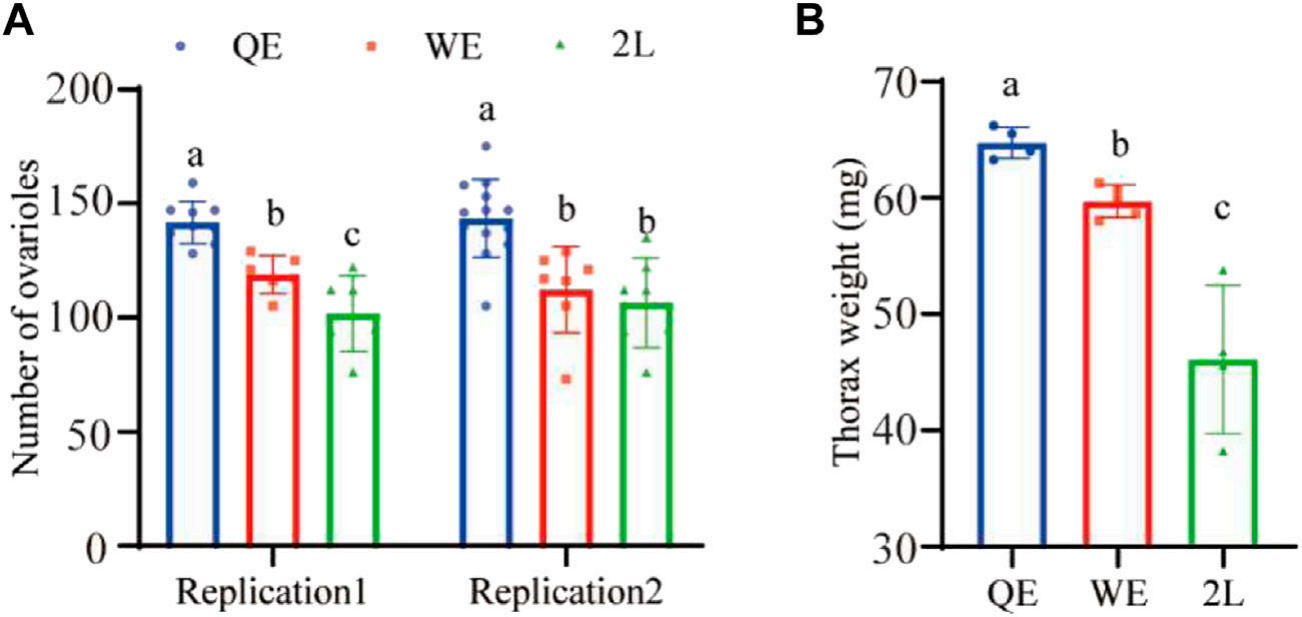
**(A)** Number of right ovarioles and **(B)** thorax weight of reared queens in the three groups (number of right ovarioles: replication 1, *n* = 9, 6, 7 for QE, WE, 2L, respectively; replication 2, *n* = 13, 7, 7 for QE, WE, 2L, respectively. Thorax weight: *n* = 4, 5, 4 for QE, WE, 2L, respectively). The bars indicate mean ± SD (standard deviation). The different letters above the bars indicate significant differences between groups (*p* < 0.05, one-way ANOVA followed by Fisher’s LSD test).

We found that the number of ovarioles (Figure 2A; replication 1: *F*_2,18_ = 21.951, *p* < 0.001; replication 2: *F*_2,24_ = 11.974, *p* < 0.001) were significantly higher in QE than in WE and 2L. Moreover, the thorax weight (Figure 2B; *F*_2,10_ = 27.728, *p* < 0.001) of queens was also significantly higher in QE than in WE and 2L and was significantly higher in WE than in 2L.

### 3.2 Reproductive quality of reared queens

**FIGURE 3:**
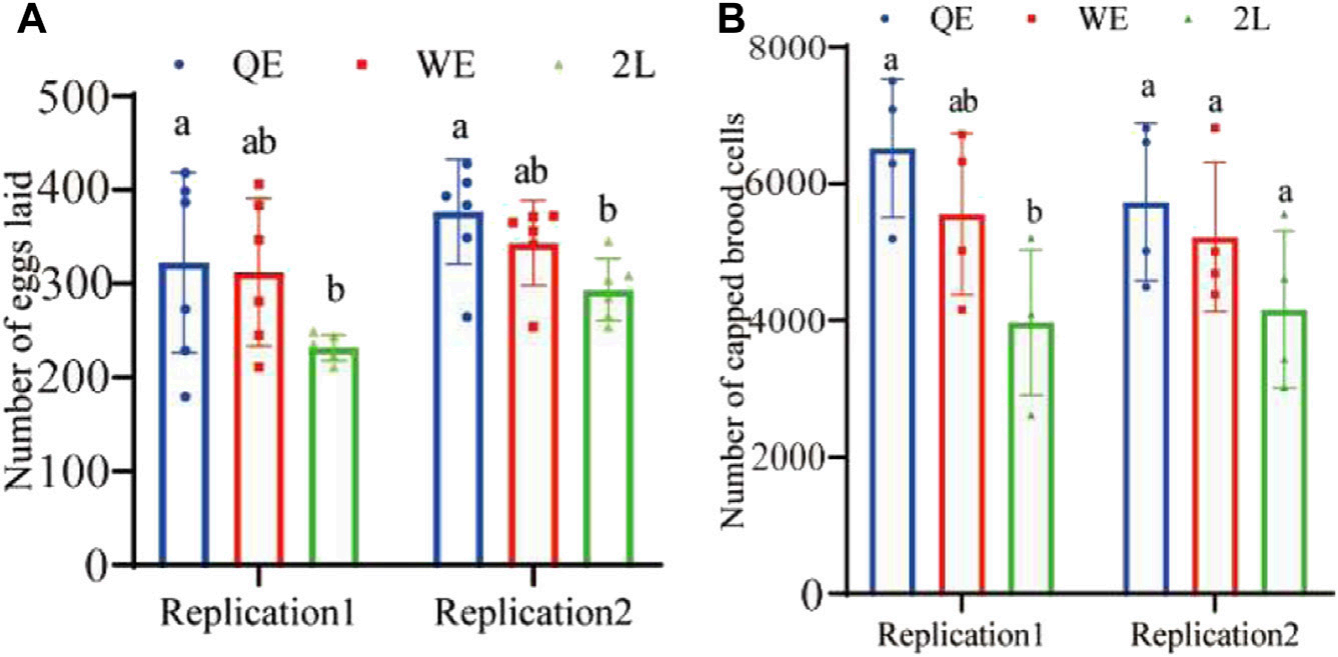
**(A)** Number of eggs laid by reared queens within 6 h and **(B)** number of capped brood cells counted during a 12-day period in each group (number of eggs laid: *n* = 6 for QE, WE, 2L in replication 1 and replication 2. Number of capped brood cells: *n* = 4 for QE, WE, 2L in replication 1 and replication 2).

The results indicated that the number of eggs (Figure 3A; replication 1: *F*_2,15_ = 2.866, *p* = 0.088; replication 2: *F*_2,15_ = 5.048, *p* < 0.05) laid by reared queens in QE was significantly higher than that in the 2L group; however, no significant difference was observed between QE and WE or between WE and 2L. Furthermore, we compared the colony growth potential of newly reared queens from QE, WE, and 2L by counting capped brood cells over a period of 12 days. The result showed that the number of capped brood cells was consistent with the results obtained for egg-laying ability. We found in replication 1, the number of capped brood cells of the QE colony was significantly higher than that in 2L. However, no significant differences were observed between QE and WE or between WE and 2L (Figure 3B; replication 1: *F*_2,9_ = 5.612, *p* < 0.05). In addition, no statistical differences were observed among the three groups in replication 2 (*F*_2,9_ = 2.010, *p* = 0.190).

### 3.3 Size of eggs laid by reared queens in the three groups

**FIGURE 4:**
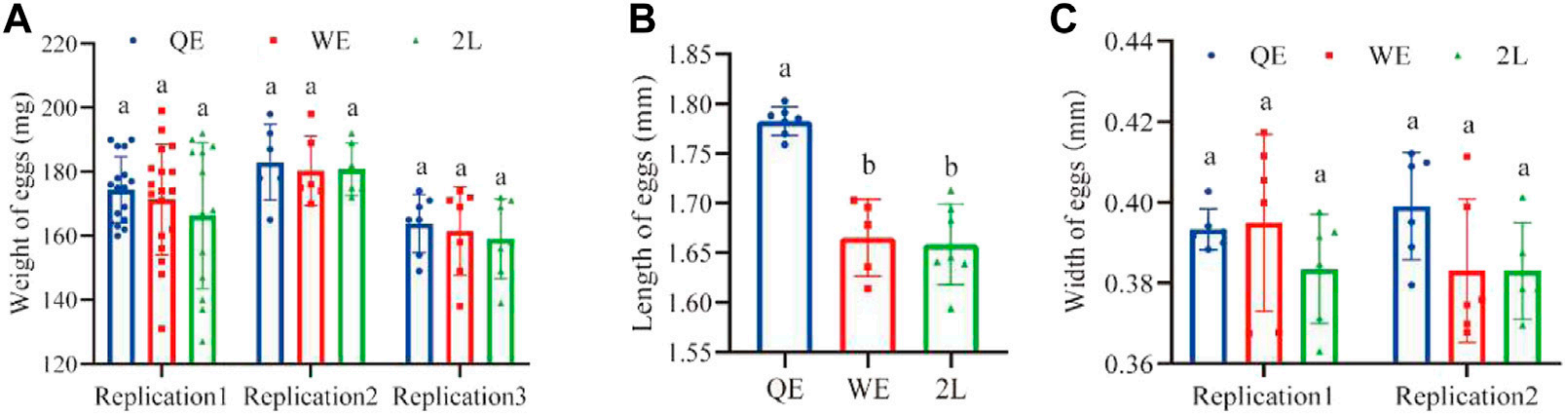
**(A)** The weight, **(B)** length, and **(C)** width of eggs laid by reared queens within the three groups (weight of eggs: replication 1, *n* = 16, 18, 13 for QE, WE, 2L, respectively; replication 2, *n* = 5, 6, 6 for QE, WE, 2L, respectively; replication 3, *n* = 7 for QE, WE, 2L. Length of eggs: *n* = 7, 5, 7 for QE, WE, 2L. Width of eggs: replication 1, *n* = 6, 6, 6 for QE, WE, 2L, respectively; replication 2, *n* = 6, 6, 5 for QE, WE, 2L, respectively).

The lengths of eggs (Figure 4B; *F*_2,16_ = 30.441, *p* < 0.001) laid by QE queens were significantly greater when compared to those in the WE and 2L groups. However, there were no significant differences in the weight (Figure 4A; replication 1: *F*_2,45_ = 0.839, *p* = 0.439; replication 2: *F*_2,14_ = 0.044, *p* = 0.957; replication 3: *F*_2,18_ = 0.274, *p* = 0.764) or width of eggs (Figure 4C; replication 1: *F*_2,15_ = 1.001, *p* = 0.391; replication 2: *F*_2,14_ = 2.298, *p* = 0.137) among the three groups.

### 3.4 Morphology and production capacity of offspring worker bees

**FIGURE 5:**
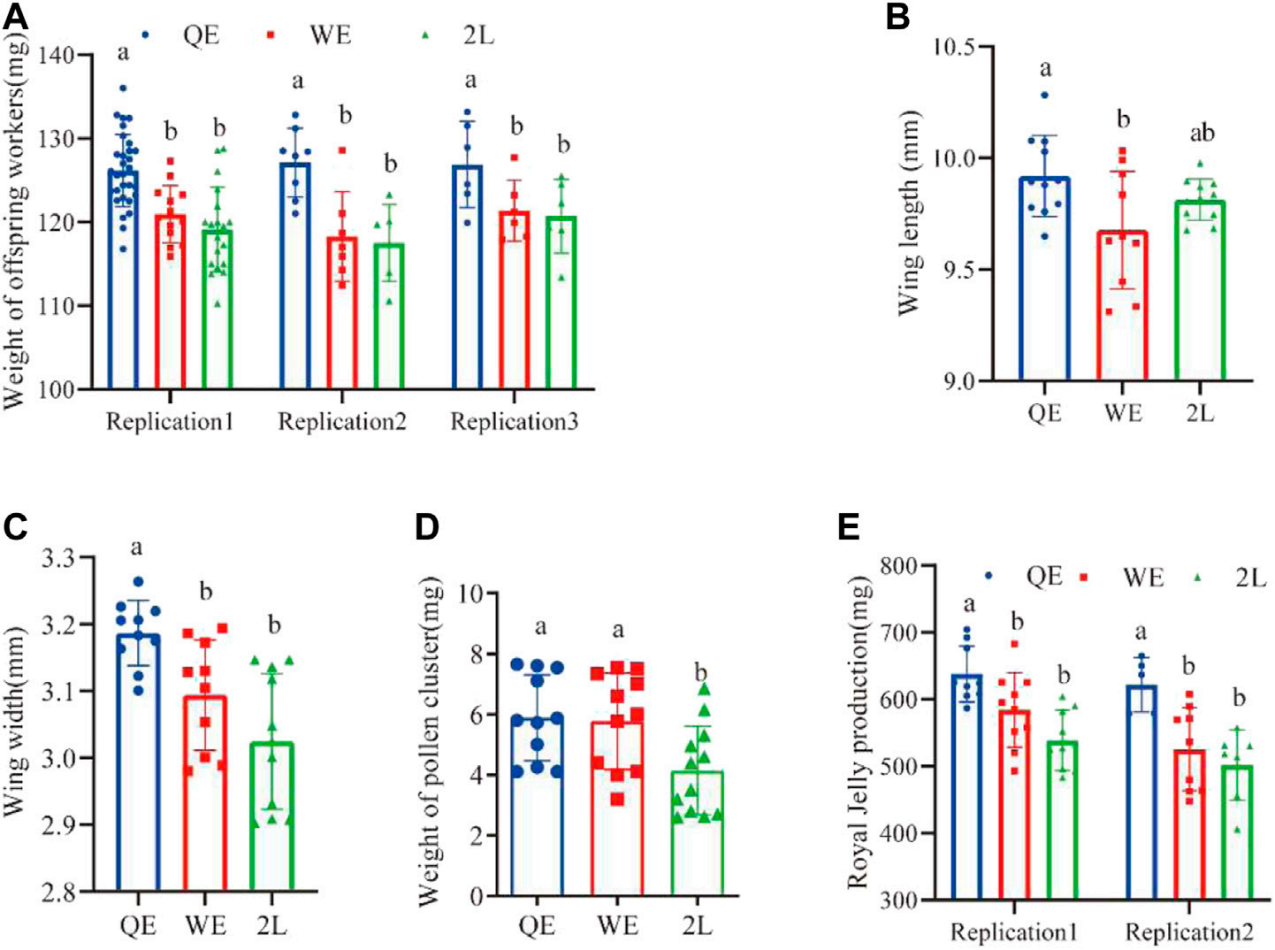
**(A)** Weight of newly emerged offspring worker bees, **(B)** wing length, **(C)** wing width, **(D)** weight of pollen on legs, and **(E)** royal jelly production (weight of newly emerged worker bees: replication 1, *n* = 31, 14, 19 for QE, WE, 2L, respectively; replication 2, *n* = 8, 7, 6 for QE, WE, 2L, respectively; replication 3, *n* = 6, 5, 6 for QE, WE, 2L. Wing length: *n* = 11, 10, 11 for QE, WE, 2L. Wing width: *n* = 10, 10, 11 for QE, WE, 2L, respectively. Weight of pollen cluster: *n* = 11, 11, 12 for QE, WE, 2L, respectively. Royal jelly production replication 1, *n* = 9, 10, 9 for QE, WE, 2L, respectively; replication 2, *n* = 5, 9, 7 for QE, WE, 2L, respectively).

Weight of the offspring worker bees from QE queens were significantly heavier than those from WE queens and 2L queens. However, there was no significant difference between WE and 2L (Figure 5A; replication 1: *F*_2,60_ = 16.891, *p* < 0.001; replication 2: *F*_2,18_ = 9.606, *p* < 0.001; replication 3: *F*_2,14_ = 4.612, *p* < 0.05). In addition, the weight of pollen on the legs of forager bees in QE and WE was significantly higher than that in the 2L group (Figure 5D; *F*_2,31_ = 4.952, *p* < 0.05). No significant differences were observed between QE and WE. The wing lengths (Figure 5B; *F*_2,29_ = 4.294, *p* < 0.05) of forager bees, as shown in Figure 3E, were significantly longer in the QE group than in the WE group, with no significant differences observed between QE and 2L or between WE and 2L. Additionally, wings were significantly wider in QE than in WE and 2L, with no significant difference between WE and 2L (Figure 5C; *F*_2,28_ = 10.360, *p* < 0.001). We also found that the production of royal jelly (Figure 5E; replication 1: *F*_2,25_ = 9.439, *p* < 0.05; replication 2: *F*_2,18_ = 7.631, *p* < 0.05) in the QE group was higher than that in WE and 2L, with no significant difference between WE and 2L.

### 3.5 Thorax size of offspring queens

**FIGURE 6:**
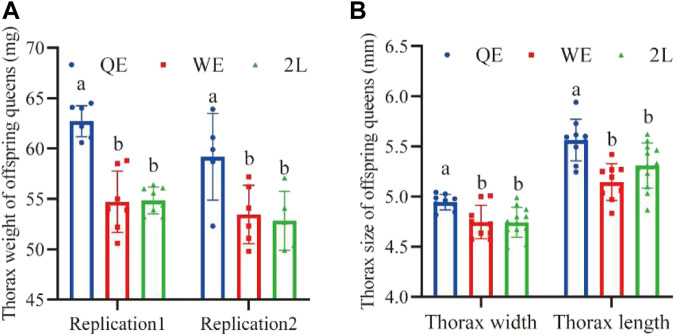
**(A)** Thorax weight and **(B)** thorax width and length of newly emerged offspring queens from each group (thorax weight: replication 1, *n* = 7, 7, 8 for QE, WE, 2L, respectively; replication 2, *n* = 5, 6, 5 for QE, WE, 2L, respectively. Thorax width: *n* = 9, 9, 12 for QE, WE, 2L, respectively; Thorax length: *n* = 9, 9, 12 for QE, WE, 2L, respectively).

We found that the thorax weight (Figure 6A; replication 1: *F*_2,19_ = 34.726, *p* < 0.001; replication 2: *F*_2,13_ = 5.397, *p* < 0.05), width (Figure 5B; *F*_2,27_ = 6.704, *p* < 0.005), and length (Figure 6B; *F*_2,27_ = 9.225, *p* < 0.001) of newly emerged offspring queens from the QE group were significantly higher than those in the WE and 2L groups; however, no significant difference was observed between WE and 2L.

## 4 Discussion

Many conditions during larval development and rearing management result in substantial differences in queen development (Wei et al., 2019; Yi et al., 2020; He et al., 2021). Research has shown that honey bee maternal effects could contribute significantly to offspring queen development (Wei et al., 2019; He et al., 2021). The results of this study demonstrated that maternal effects not only increased the quality of queens but also strongly influenced the reproductive ability of offspring queens as well as the foraging capacity of offspring worker bees. These findings indicate that honey bee maternal effects can improve the fitness of the whole offspring colony.

Our results are consistent with those of our previous study indicating that offspring from QE demonstrated significantly higher weights and thorax width than those from both WE and 2L (Yu et al., 2022). Furthermore, the expression levels of development- and reproduction-related genes of offspring queens from QE queens were higher than those of both WE and 2L (Wei et al., 2019; Yu et al., 2022). QE queens also had an improved reproductive ability, such as more and larger eggs, in worker cells than those from the WE and 2L groups (Figures 2–4), resulting in larger offspring workers (Figures 5A–C). These findings revealed that honey bee maternal effects play an important role in queen development and can directly impact reproductive ability. The growth, productivity, and survival of a honey bee colony is highly dependent on the health and reproductive capacity of its queen (Nelson and Gary, 1983; Rangel et al., 2013; Tarpy et al., 2013). It has been reported that the initial weight and thorax size are strongly correlated with the queen ovariole number, and they influence the quality of queen (Nelson and Gary, 1983; Dodologlu and Gene, 2003; Amiri et al., 2017; Wei et al., 2019; He et al., 2021). Therefore, improving honey bee queen quality via maternal effects would be beneficial for the apicultural industry, especially in the context of the current threats to honey bee production, such as parasites and pesticides, and global reductions in colonies (Hatch et al., 1999; Kirsten et al., 2012; Amiri et al., 2017).

Interestingly, the results of this study indicate that honey bee maternal effects could be directly transferred to subsequent generations. We found that this effect strongly influenced the reproductive potential of offspring queens and the working performance of offspring workers (Figures 5, 6). The pollen-collecting and royal jelly-producing capacities of the QE offspring workers were also higher than those of workers from WE and 2L (Figures 5D, E). QE offspring queens also had higher birth weights, lager thorax sizes, and more ovarioles than those of offspring queens from WE and 2L (Figure 6) (Yu et al., 2022). These findings suggest that honey bee maternal effects have profound impacts on the offspring phenotype (Wolf and Wade, 2009; Wei et al., 2019). Furthermore, the cross-generational effect could improve the fitness of the whole colony. We found that QE offspring workers had larger body and wing sizes, collected larger pollen balls, and produced more royal jelly (Figure 5). This not only directly increases the productivity of a honey bee colony but is also an indicator of the health status and survival thereof (Stankus, 2008; Ratnieks and Carreck, 2010; van Engelsdorp et al., 2011; Lee et al., 2019). Moreover, the thorax size of offspring queens from QE was higher than that of offspring queens from WE and 2L (Figure 6). Thus, the heavier and larger QE offspring queens in this study may be correlated with high reproductive potential.

Our results indicated that honey bee maternal effects directly influence queen quality and the effects can be transmitted to later generations. As an evolutionary strategy, this maternal effect has likely contributed to honey bee adaptation to environmental changes. However, domestic queen rearing practices hinder this maternal effect and have long-term deleterious effects. Alternatively, commercial rearing of honey bee queens from eggs laid in queen cells should be widely used in the beekeeping industry.

## Data Availability

The raw data supporting the conclusion of this article will be made available by the authors, without undue reservation.
